# Five Novel Non-Sialic Acid-Like Scaffolds Inhibit In Vitro H1N1 and H5N2 Neuraminidase Activity of Influenza a Virus

**DOI:** 10.3390/molecules25184248

**Published:** 2020-09-16

**Authors:** Luis Márquez-Domínguez, Julio Reyes-Leyva, Irma Herrera-Camacho, Gerardo Santos-López, Thomas Scior

**Affiliations:** 1 Laboratorio de Virología, Centro de Investigación Biomédica de Oriente, Instituto Mexicano del Seguro Social, Metepec, Puebla 74630, Mexico; lumardo80@gmail.com (L.M.-D.); reyesleyva@yahoo.com (J.R.-L.); 2 Posgrado en Ciencias Químicas, Benemérita Universidad Autónoma de Puebla, Puebla 72570, Mexico; 3 Laboratorio de Simulaciones Computacionales Moleculares, Facultad de Ciencias Químicas, Benemérita Universidad Autónoma de Puebla, Puebla 72592, Mexico; 4 Centro de Química, Instituto de Ciencias, Benemérita Universidad Autónoma de Puebla, Puebla 72570, Mexico; irma.herrera@correo.buap.mx

**Keywords:** influenza, flu drugs, neuraminidase inhibitors, noncompetitive inhibition, scaffold hopping, ligand docking

## Abstract

Neuraminidase (NA) of influenza viruses enables the virus to access the cell membrane. It degrades the sialic acid contained in extracellular mucin. Later, it is responsible for releasing newly formed virions from the membrane of infected cells. Both processes become key functions within the viral cycle. Therefore, it is a therapeutic target for research of the new antiviral agents. Structure–activity relationships studies have revealed which are the important functional groups for the receptor–ligand interaction. Influenza virus type A NA activity was inhibited by five scaffolds without structural resemblance to sialic acid. Intending small organic compound repositioning along with drug repurposing, this study combined in silico simulations of ligand docking into the known binding site of NA, along with in vitro bioassays. The five proposed scaffolds are *N*-acetylphenylalanylmethionine, propanoic 3-[(2,5-dimethylphenyl) carbamoyl]-2-(piperazin-1-yl) acid, 3-(propylaminosulfonyl)-4-chlorobenzoic acid, ascorbic acid (vitamin C), and 4-(dipropylsulfamoyl) benzoic acid (probenecid). Their half maximal inhibitory concentration (IC_50_) was determined through fluorometry. An acidic reagent 2′-*O*-(4-methylumbelliferyl)-α-d*N*-acetylneuraminic acid (MUNANA) was used as substrate for viruses of human influenza H1N1 or avian influenza H5N2. Inhibition was observed in millimolar ranges in a concentration-dependent manner. The IC_50_ values of the five proposed scaffolds ranged from 6.4 to 73 mM. The values reflect a significant affinity difference with respect to the reference drug zanamivir (*p* < 0.001). Two compounds (*N*-acetyl dipeptide and 4-substituted benzoic acid) clearly showed competitive mechanisms, whereas ascorbic acid reflected non-competitive kinetics. The five small organic molecules constitute five different scaffolds with moderate NA affinities. They are proposed as lead compounds for developing new NA inhibitors which are not analogous to sialic acid.

## 1. Introduction

Influenza is a severe and infectious respiratory disease with high mortality and morbidity rates. Two subtypes of influenza A viruses (H1N1 and H3N2) along with two lineages of influenza B viruses (Victoria and Yamagata) are cocirculating in the human population. The World Health Organization (WHO) estimates that there are approximately 290,000 to 600,000 annual deaths worldwide associated with the infection [1,2]. Both influenza viruses A and B possess two surface glycoproteins, hemagglutinin (HA) and neuraminidase (NA) [3,4]. Both glycoproteins have complementary roles within the viral cycle, despite the fact that both recognize the same molecule in infected host cells, namely the terminal sialic acid monomers on oligomeric glycoconjugates [5,6].

NA mediates a pivotal step in the life cycle of influenza A viruses and thereby contributes to their transmissibility and pathogenicity—all of which characterizes it as a potential viral protein target. Commercial drugs against this infectious disease in oral dosage forms have been derived from the aforementioned terminal sialic acid which constitutes the natural substrate of NA [7,8]. Precisely, NA belongs to the third enzyme class of hydrolases (EC 3.2.1.18) and catalyzes the cleavage of α-ketosidic bonds between N-acetylneuraminic acids and adjacent glycol conjugate carbohydrates [9,10]. The enzyme is classified into eleven subtypes (N1 to N11). They are divided into two genetically different groups. N1, N4, N5 and N8 belong to Group-1, whereas N2, N3, N6, N7 and N9 form Group-2 [11,12]. The distinction matters for ligand docking studies because both groups show observed conformational differences in a certain loop segment of their crystal structures at the active site. This loop is identified by residues 148-151. In Group-1 neuraminidases it presents an “open” conformational state leaving some extra space for ligand occupation (additional cavity or pocket). Upon inhibitor binding the loop closes down on this additional binding pocket (loop residue 150). In stark contrast, Group-2 neuraminidases always adopt a “closed” conformation, regardless of the presence or absence of a ligand at the active site. As a direct consequence, a distinction between liganded and unliganded (apo-form) has to be made for Group-1 receptor docking.

Changes in the primary sequence have been detected and may reflect drug resistance phenomena, in particular, residue mutation patterns at the binding sites of both neuraminidase groups [13]. In a more general view, the antigenic shifts of influenza viruses are a threat to public health care and infectious disease prevention. Continuous vaccine turnover has become mandatory when trying to establish effective preventive vaccination campaigns to protect the human population worldwide [14]. Over time, the primary sequence variations have also induced failures in the effectiveness of first line oral drug amantadine.

Nevertheless, oral drug administration seemingly shortens the duration of disease or convalescence in addition to reducing the risk of complications or the patient’s death if the drug treatment is applied on time. Very early during the initial stages of infection, neuraminidase inhibitors have a dual effect not only as a causal cure attacking the infection cycle but also as a prophylactic tool against influenza virus infections. In recent years, emerging single or multidrug-resistant strains have threatened the therapeutic achievements. Several mutational changes have been associated with drug resistance [15]. In 2018, Toledo and coworkers reported a large number of mutations in the primary sequences of NA taken from patient samples in Mexico, which reflects the relatedness between amino acid changes and drug resistance. [15]. One could reason that the particularly inadequate use of oseltamivir in Mexico—without proper medical diagnosis and prescription or professional treatment supervision (pharmaceutical care taking)—could have imposed evolutionary pressure on the influenza A virus population allowing only oseltamivir-resistant strains to survive. In the past, pharmaceutical companies have intensified research as an answer to the pharmacotherapeutic challenges and introduced new NA inhibitors like zanamivir [7,8,16].

To this regard the present study aims at proposing non-sialic acid-like scaffolds with proven binding site affinity to address the drug resistance problem suggesting the development of scaffold derivatives to enhance binding site affinity to mutated neuraminidases.

## 2. Results

### 2.1. Analysis of the Drug-Like Structures

**Figure 1:**
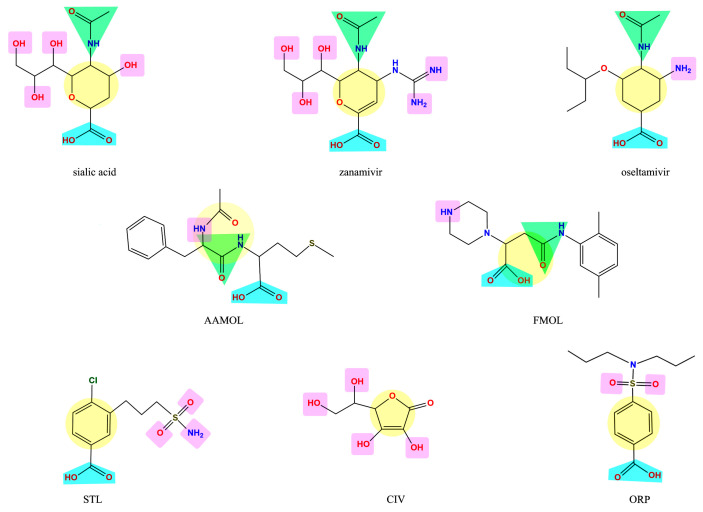
Display of structural similarities between sialic acid and two neuraminidase inhibitors (reference drugs) and the five proposed scaffolds. The structural similarities are coded by colored shapes: yellow circles—the aliphatic ring; blue trapezoids—the carboxyl group; green triangles—the amide group; purple squares—the groups that have capacity to form hydrogen bridges.

The molecules under scrutiny do not represent analogues of neuraminidase inhibitors oseltamivir and zanamivir—albeit to some extent they are structurally related to natural substrate sialic acid by a set of common features (Figure 1). Four drug-like compounds (FMOL, AAMOL, STL and ORP) bear an aromatic ring as an additional feature. In ORP it even replaces the sialic core. In addition to the intended drug repurposing of ORP, we included vitamin C (CIV). ORP was introduced to the drug market as an oral uricosuric drug (gout). CIV was selected for three reasons: (i) its surprising structural similarity in its ring-closed form (lactone); (ii) its wide-spread oral administration as either “anti-flu medicine” or popular co-medication, and (iii) the poor bibliographic evidence about its antiviral activities.

It is noteworthy to explain that the internal acronyms reflect certain features of the five studied molecules as potential NA inhibitors: *N*-acetylphenylalanylmethionine was named AAMOL because it constitutes a dipeptide; hence “AA” symbolizes two amino acids. FMOL is the short name of propanoic 3-[(2,5-dimethylphenyl) carbamoyl]-2-(piperazin-1-yl) acid, and alludes to the first introduction of an aromatic ring. The phenyl group was written with an “F” like the one-letter code “F” for phenylalanine or “fenil” in Spanish and therefore the scaffold was called FMOL. The “S” in STL reflects the sulfonamide class of substance: 3-(propylaminosulfonyl)-4-chlorobenzoic acid. The two repositioned or repurposed molecules were ascorbic acid (common name: vitamin C) and 4-(dipropylsulfamoyl) benzoic acid (INN drug name: probenecid). CIV and ORP are their respective inverted code names. Thanks to their coded labels, all substances could be tested in the laboratory without user bias.

### 2.2. Analysis of the Amino Acid Sequences of Influenza a Viruses

As input data for our studies we included two influenza strains: (i) the human A/PR/8/34 (H1N1) and (ii) the avian A/Chicken/Mexico/31381-7/1994 (H5N2). Multiple sequence alignments (MSA) were performed with different sequences of influenza A neuraminidases (from N1 to N9) to identify segments of conserved amino acids which correspond to residues at the active site. Residues R118, D151, R152, R224, E276, R292, R371 and Y406 constitute the catalytically active residues within the binding site. They interact directly with the substrate, sialic acid (SA). Residues E119, R156, W178, S179, D198, I222, E227, H274, E277, N294 and E425 assist the recognition and binding of ligands [17,18,19]. MSA studies identified conserved residues R118, E119, R152, R156, R224, E227, H274, E276, E277, R292, N294 and R371 in almost all subtypes of neuraminidase [11,12,17,18,19].

### 2.3. Molecular Docking

**Figure 2:**
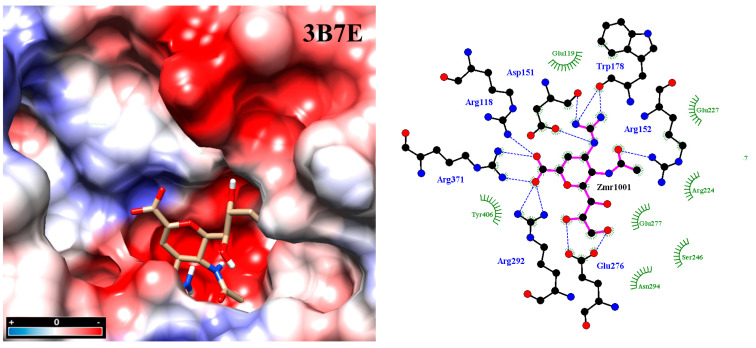
Molecular docking between zanamivir molecule with neuraminidase subtype N1 3B7E. Intensive hydrogen bond networking helps stabilize inhibitor binding at the active site, in addition to hydrophobic interactions. Analysis was carried out with the program PrPx-Phyton v0.8 (Autodock Vina). The 3D model surface was colored by the Coulombic Surface tool under UCSF Chimera 1.11.2. LigPlot v.1.4.5 analyzed the final docked poses. Dotted blue lines symbolize hydrogen bonding while semicircles with tabs show hydrophobic interactions.

Molecular docking was carried out under Autodock Vina [20,21] using two crystal structures of neuraminidase: A/Brevig Mission/1/1918 H1N1 (PDB code: 3B7E [22]), as well as A/Tanzania/205/2010 H3N2 (PDB code: 4GZW [23]). All conformations with the lowest interaction energy were analyzed with LigPlot v.1.4.5 and displayed in a 2D interaction map (Figure 2) [24]. Inspecting the final docked poses helped identify typical noncovalent interaction patterns such as hydrogen bonds, ionic or saline bridges, van der Waals or hydrophobic interactions, all of which are commonly seen in reversible drug-receptor complexes. Not all observed drug-interacting residues at the active site were also reported in the literature [25,26,27,28,29]. This finding constitutes a key argument in favor of the five scaffolds in view of drug resistance by mutated residues. In particular, the binding pattern varies from scaffold to scaffold according to the substituents/functional groups on each scaffold.

In the next step, we retrieved the neuraminidase sequences of human A/PR/8/34 (H1N1) strain (NCBI GenBank: ABD77678.1) and avian A/Chicken/Mexico/31381-7/1994 (H5N2) strain (NCBI GenBank: ACZ36772.1). To obtain full-length three-dimensional structures of our target proteins, we used I-TASSER for homology modeling [30,31].

**Figure 3:**
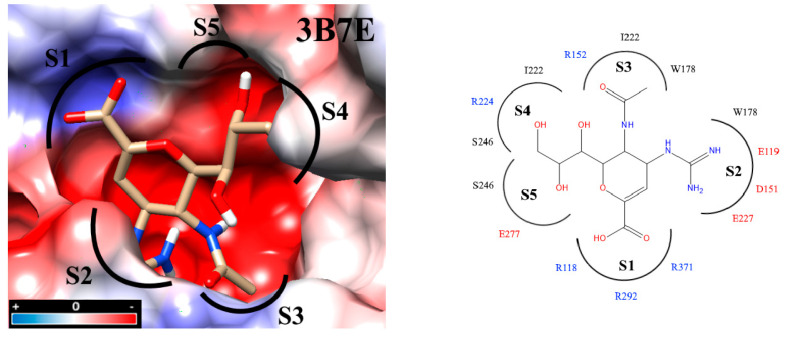
Docking inside to the active site of neuraminidase subtype N1 (PDB code: 3B7E) with molecule zanamivir. The active site is divided into five subsites (S1 to S5) and these subsites are composed of acidic (red), basic (blue) and hydrophobic (black) amino acids. The interaction analyses were carried out with PrPx-Phyton v0.8 and Autodock Vina. The colored surfaces were generated with Coulombic Surface Coloring under UCSF Chimera 1.11.2. Red resp. blue surfaces represent the regions with acidic or basic amino acids, respectively. White surfaces are hydrophobic regions. Right side panel: schematic drawing of the active site with bound zanamivir and the five subsites S1 to S5.

Interaction analyses allowed the observation of the residues that form hydrogen bonds with the ligands. These interaction analyses were based on the outcome of docking studies to determine how the ligand occupies the active site. These computed binding modes were found to be on an equal footing with our experimental results reflecting how efficient our molecules were with respect to the zanamivir control. Precisely, the active site of NA is extremely polar. To facilitate the inspection of the inhibitor binding modes, the active site was divided into five subsites (S1-S5) [27,32,33] (Figure 3). S1 is composed of three basic amino acids (R118, R292, and R371), whereas S2 is formed by three acidic amino acids (E119, D151, and E227) in addition to W178. Intriguingly, all three residues R152, W178 and I222 of the S3 subsite possess long sidechains. The crystal structures of protein–compound complexes (PDB codes: 3B7E [22], 2HU4 [34] and 1MWE [35]) informed us about how the acetamido moieties of sialic acid, zanamivir or oseltamivir could engage in hydrogen bond networking with arginine R152 of this binding subsite. The remaining subsites S4 (I222, R224 and S246) and S5 (S246 and E277) are hydrophobic in nature and were treated separately (cf. Section 2.7. Determination of IC_50_).

**Figure 4:**
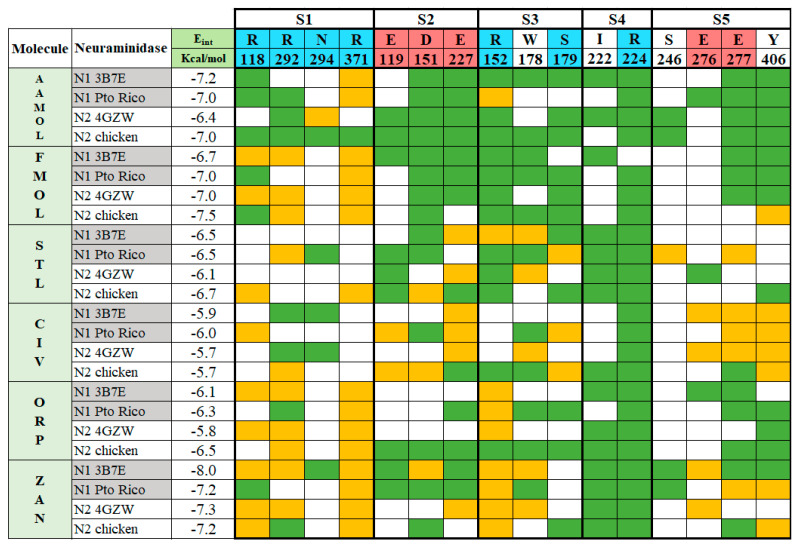
Binding pattern between the ligands AAMOL, FMOL, STL, CIV, ORP or zanamivir and the interacting amino acids of group 1 and 2 neuraminidases. The active site was subdivided into five subsites for inspection. Color code of the table cells: blue or red for positively or negatively charged interacting residues, respectively; yellow for hydrogen bonds; and green for hydrophobic contacts. The analysis was carried out with the program LigPlot v.1.4.5 for the docked poses with neuraminidases H1N1 A/PR/8/34 and H5N2 A/CHK/MX/1994 (PDB codes: 3B7E and 4GZW, respectively).

The experiments with the four neuraminidases were carried out in presence of the reference drug zanamivir. The catalytic residues within the active sites were identified as R118, R292 and R371 at subsite S1 and D151 at subsite S2, R152 at subsite S3, and E276 [9,17,36,37]. They are all interacting with zanamivir whose binding mode allows the drug to tightly attach to the viral enzyme and strongly inhibit the enzymatic activity, since the aforementioned residues are necessary for natural substrate recognition (Figure 4).

### 2.4. Analysis of Functional Substituent Groups on Zanamivir

**Figure 5:**
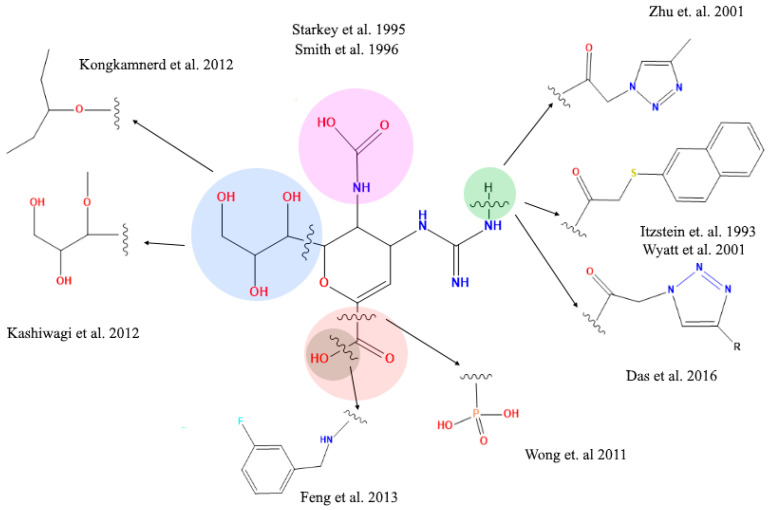
Structural modifications of zanamivir molecule reported in the literature. The amide and carboxyl groups are essential for the interaction with the arginine residues at the active site of the viral neuraminidase target protein.

The literature reports about different structural modifications of reference drug zanamivir. Starkey et al. (1995) [38] synthesized a compound without the acetamide group in position 5, generating a compound that completely lost the inhibitory activity, in contrast to Smith et al. (1996) [39] who exchanged the acetamide group in position 5 with different organic rests. This way they succeeded in generating compounds that significantly reduced the inhibitory activity. These studies have established critical aspects of how the 5-acetamide group contributes to binding affinity of zanamivir. Moreover, Shie et al. (2011) [40] replaced the carboxyl functional group for a tamiphosphor group which has more extensive hydrogen-bonding interactions with key residues on the NA active site than the carboxylate group. However, the latter functional group—together with the drug’s guanidinium group—possess an extremely high polarity which causes a poor bioavailability. Feng et al. [41] substituted the hydroxyl part of the 1-carboxyl group by benzylamines. These elongated amide groups at C-1 position resulted in direct contacts with the 430-cavity of NA under the formation of additional hydrogen bonds and strong hydrophobic interactions. Kongkamnerd et al. (2012) [42] modified the guanidino group for an amino group in position 4 and glycerol in position 6 for a pentyl ether. These changes increased lipophilicity which allows oral administration thanks improved oral bioavailability (oseltamivir). Positions 4 and 6 have been replaced by larger and more complex residues generating new compounds. Yet, they remain analogous to sialic acid and so they may suffer activity losses from the appearance of mutations within the NA’s active site in viral strains after developing resistance to such sialic acid-like antiviral drugs (Figure 5) [41,42,43,44,45,46].

The five scaffolds were proposed since they are not analogous to sialic acid. Nevertheless, they present similarities in their functional groups (Figure 1) and show affinity to the viral neuraminidase by interacting with different amino acids at the active site (Figure 4). In addition, they are amenable to structural modifications to generate molecules with higher affinity as outlined in the following.

STL and CIV reveal a diversification in their interaction profiles, i.e., they interact with different residues depending on the two NA subtypes. Especially, CIV can occupy the cavity in a multiple binding mode. Thanks to its small molecular size and volume it can interact with different residues in a variety of final poses at the active site. Like CIV, STL cannot be docked in an optimal way leading to affinity losses compared to reference drug zanamivir. Its aminosulfonyl group sidechain makes an unfavorable kink and subsequently the ligand shifts into less favorable locations. This, in turn, decreases the anchoring capacity of the molecule within the active site with respect to zanamivir.

FMOL is a typical scaffold since it is poorly decorated with functional groups considering its molecule weight. Few hydrogen bonds, polar and nonpolar interactions contribute to target affinity. Hence, it promises huge affinity improvements upon derivatization.

ORP can interact with Arg118 and Arg152, following a characteristic binding pattern that was seen in almost all analyzed neuraminidases. This finding creates the opportunity to enhance affinity by introducing side chains in the scaffold’s ring meta position, for instance -OH or -NH_2_ groups. Alternatively, the sulfonyl group can be moved to the meta position to reach other residues at the catalytic site.

### 2.5. Determination of Neuraminidase Activity

In the in vitro experiments, Madin-Darby canine kidney (MDCK) cell lines were infected with human influenza A H1N1 and avian H5N2 viruses. The supernatants were collected by observing an extensive cytopathic effect at 96 h post-infection (hpi). The supernatants were stored in aliquots at −70 °C for the determination of enzymatic activities of the neuraminidase and viral titration by means of lytic plaques [47,48].

The enzymatic activity of NA was analyzed with the fluorescent substrate MUNANA. The standardized assay allowed us to compare data between different viral isolates and assays between different laboratories. Samples of cell supernatant infected with whole virus were used as source of enzymes. The numbers of either virions in the samples or the numbers of NA molecules in each virion may vary from strain to strain. Hence, we used the criteria reported by Marathe et al. in 2013 to select the amount of enzyme or viral dilution to provide adequate fluorescence for our trials. The relative fluorescence units (RFU) used in our assays ranged from 15,000 to 45,000 RFU. In this range no saturation of the system occurred and linearity existed in the quantification method.

In addition, serial 1:2 dilutions were prepared from the same supernatants and it was observed that dilutions generated a proportional increase in the concentration of 4-methylumberiferone with respect to the incubation time for MUNANA substrate. This observation attests that the dilutions generated a linear increase in the fluorescent product with respect to time [49].

### 2.6. Determination of Kinetic Parameters

**Figure 6:**
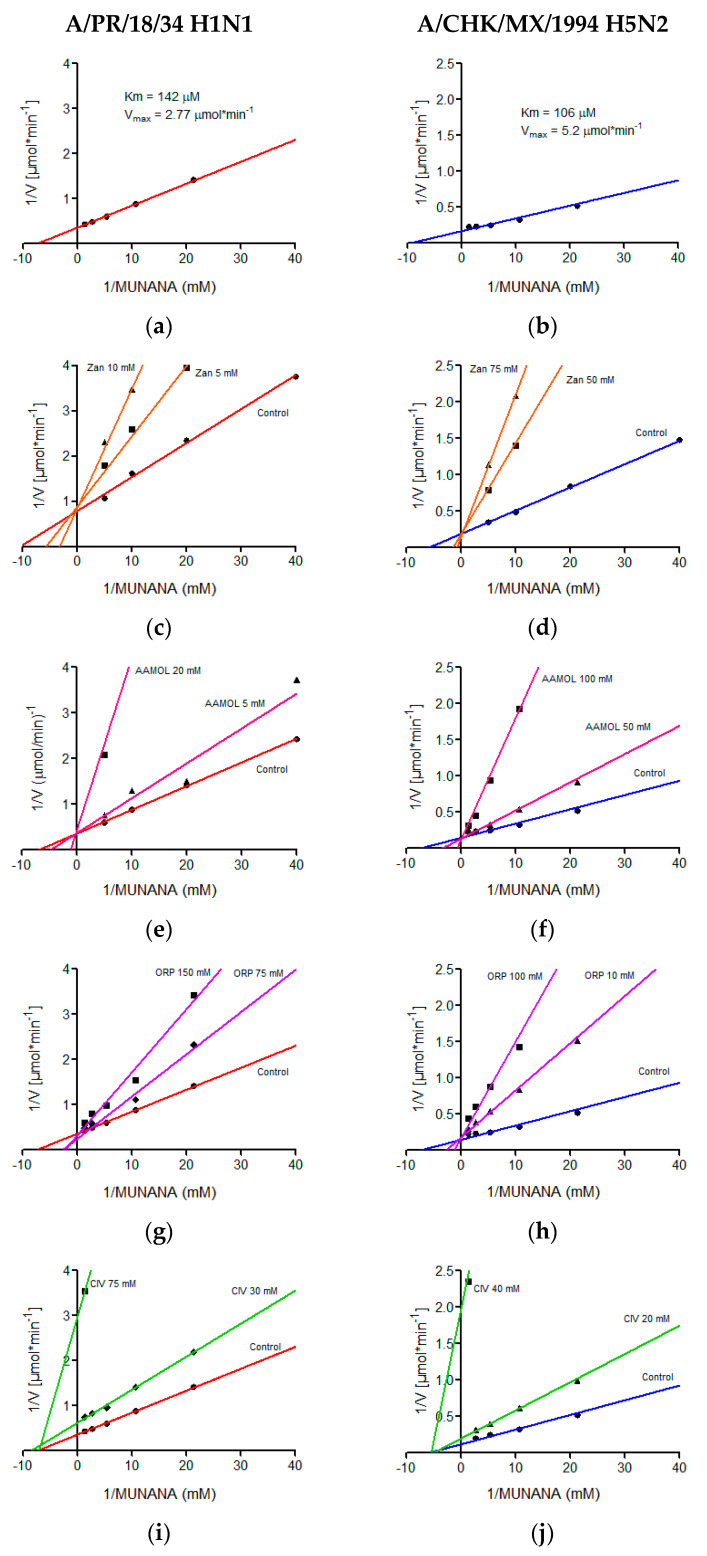
Lineweaver-Burk diagrams of two influenza A virus strains. Panels (**a**), (**c**), (**e**), (**g**) and (**i**) report the kinetic analyses of viral strain A/PR/18/34 H1N1, while panels (**b**), (**d**), (**f**), (**h**) and (**j**) report the kinetic analyses of viral strain A/CHK/MX/1994 H5N2. Panels (**a**) and (**b**) show treatments with MUNANA as a substrate. Panels (**c**) and (**d**) present treatments with zanamivir; panels (**e**) and (**f**) with AAMOL; panels (**g**) and (**h**) with ORP; and panels (**i**) and (**j**) with CIV, respectively. The GraphPadPrism 6.04 program was used to determine values of the inhibition constant (Ki) for the molecules analyzed.

The kinetic parameters of the H5N2 and H1N1 avian influenza viruses were determined in a MUNANA concentration range of 0.02 mM to 0.75 mM, demonstrating typical Michaelis-Menten kinetics, which was graphed by the double reciprocal, better known as the Lineweaver-Burk plot where the Km value of 106 μM and a Vmax of 5.2 μmol min^−1^ were calculated for the avian virus and for the human being a Km of 142 μM and a Vmax of 2.77 μmol min^−1^ (Figure 6). We quantitatively determined the in vitro effects of AAMOL, CIV and ORP on the enzyme activity of the viral neuraminidase from two virus strains, namely (i) A/Puerto Rico/8/34 Influenza A Virus subtype H1N1 (A/PR/8/34 H1N1) and (ii) A/chicken/Mexico/232/94 Influenza A Virus subtype H5N2 (A/CHK/MX/94 H5N2). To this end, an inhibition test was carried out using MUNANA as substrate. At that stage, the experimental data were registered to construct the corresponding diagram of Lineweaver-Burk plotting 1/V (reciprocal of the reaction rate) against 1/[S] (reciprocal of substrate concentration).

Our inhibition control (zanamivir), as well as test substances AAMOL and ORP, showed a competitive inhibition mechanism for both NA subtypes. In this type of mechanism, the substrate and the inhibitor are exclusive, which indicates that only one of them can occupy the active site of viral neuraminidase (Figure 6).

For the CIV molecule, the type of inhibition mechanism was characterized as being noncompetitive in nature. Henceforth, the inhibitor CIV succeeded in bind to neuraminidase, but did not modify its affinity for the substrate. It modified, however, the enzymatic velocity. For this type of mechanism, the inhibitor could bind to the enzyme forming the complex E-I (enzyme-inhibitor) or the tertiary complex E-S-I (enzyme-substrate-inhibitor). Here substrate and inhibitor are no longer mutually exclusive (Figure 6). Another explanation would be the formation of covalent bonds (cf. Section 2.8. Inhibition of viral replication).

AAMOL, ORP and CIV inhibit the enzymatic activity in the order of the millimolar, however, these structures can be modified, adding polar residues that allow us to increase the affinity of neuraminidase for these and decrease the value of its inhibition constant, Ki, allowing the occurrence of an inhibitory action with lower concentrations of inhibitor (Table 1).

### 2.7. Determination of Half Maximal Inhibitory Concentration (IC_50_)

**Figure 7:**
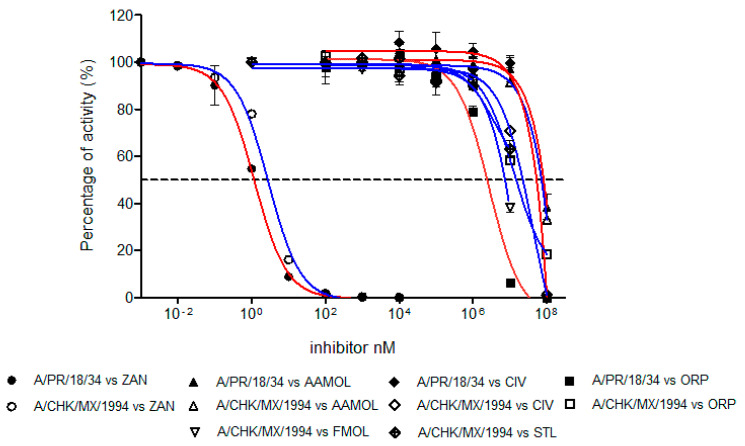
Concentration-activity diagram for the viral supernatant which was used along with the inhibition control zanamivir. They were prepared with 0.3 mM MES buffer and 4 mM CaCl2 at pH 6.5. FMOL and STL molecules were evaluated for H5N2 avian influenza virus while other molecules and the control were evaluated with both viral subtypes: human H1N1 and avian H5N2. The IC_50_ of each curve was graphed and determined with the GraphPad Prism 6.04 program. Nonlinear regression analysis was performed to obtain trend lines. Data represent the average ± SD of three independent experiments. The values obtained from the IC_50_ are reported in Table 1.

The inhibition strength of the enzymatic activity of influenza A virus neuraminidase was determined and measured as IC_50_ values for the human H1N1 and avian H5N2 viruses using zanamivir as a positive control of the inhibition (Figure 7). The IC_50_ value of zanamivir for the human H1N1 subtype was 1.19 nM whereas for the avian H5N2 subtype it yielded a value of 2.84 nM. These values lie within the reported range for other neuraminidases of different influenza A subtypes [47,49,50]. This way we validated our assay method.

The inhibition activities of AAMOL, FMOL, STL, CIV and ORP were assessed for the two NA targets from both, the avian influenza A virus subtype H5N2 as well as the human H1N1. All IC_50_ values lay in the millimolar range (Figure 7, Table 1).

The difference between the tested inhibition concentrations and the reference concentration of zanamivir indicated that all five proposed scaffolds possess much lower affinities to neuraminidase. This comes, however, as no surprise as we designed scaffolds and not structurally elaborated drug candidates.

Scaffold ORP differs significantly in its avian and human subtype IC_50_ values. At first sight, the observation was quite annoying because the binding models for both subtypes maintain a common interaction pattern. A more detailed inspection of Autodock Vina`s calculated docked poses solved the apparent prediction failure at an atomic scale. We detected major hydrophobic contributions to the ligand-receptor binding in the cavity of subtype N1. The discrepancy between observed and calculated data originates in the physical nature of those nonpolar group-to-nonpolar group interactions. Those quantities are crude estimates based on linear scaling of some computable features, but other fundamental processes (e.g., bulk water versus hydration shell, ordered solvent or solution entropy etc.) remain unaccounted for in molecular mechanics calculations of docking simulations. Autodock Vina was released as a direct answer to the manifold (cryptic) parameter settings of Autodock 4.2 with their need for a “case-to-case fine tuning” (user calibration) to run Autodock 4.2. Back then, hydrophobic forces like entropic and solvent effects had been integrated into the docking force field by empiric calibration of published ligand–receptor training data and averaged to fit test set examples under a linear scaling approach. A trade-off between larger training sets—to treat more of the binding site and mode variations to widen the applicability range to gain user popularity—and reproducibility (predictability) concerns had to be made by the program developers. Bearing this in mind now, it becomes quite logical to experience this prediction failure with subtype N1 where the important gain in hydrophobic forces remained unaccounted for. In more general terms, empiric parameters describe hydrophobic contributions to docking energies which is a necessary over-simplification of the physicochemical first principles unless a quantum chemistry approach is used. This type of docking pitfalls was more frequent with Autodock 3 which exaggerated hydrogen bonding contributions to the final docking energy term (ligand affinity) and triggered a new release (Autodock 4) for treatment of proteins with extended hydrophobic regions like the hydrophobic subsites S4 (I222, R224 and S246) and S5 (S246 and E277).

### 2.8. Inhibition of Viral Replication

**Figure 8:**
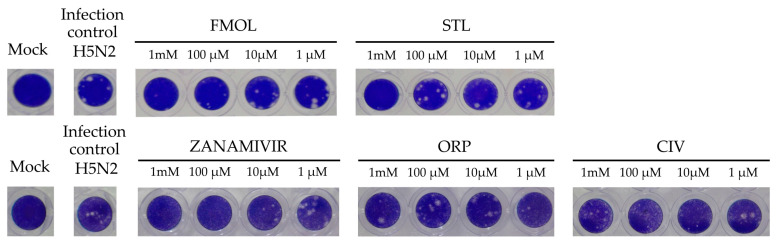
Display of the lytic plaques for inhibition of viral replication. The screening test was carried out for FMOL, STL, CIV, ORP and zanamivir as inhibition control in Madin–Darby canine kidney (MDCK) cells and infecting the avian influenza A A/CHK/MX/1994 H5N2 virus. The number of plaques was counted, and the percentage of activity was calculated by comparing treated versus untreated cells. Of note, the experiment for AAMOL was not carried out.

**Figure 9:**
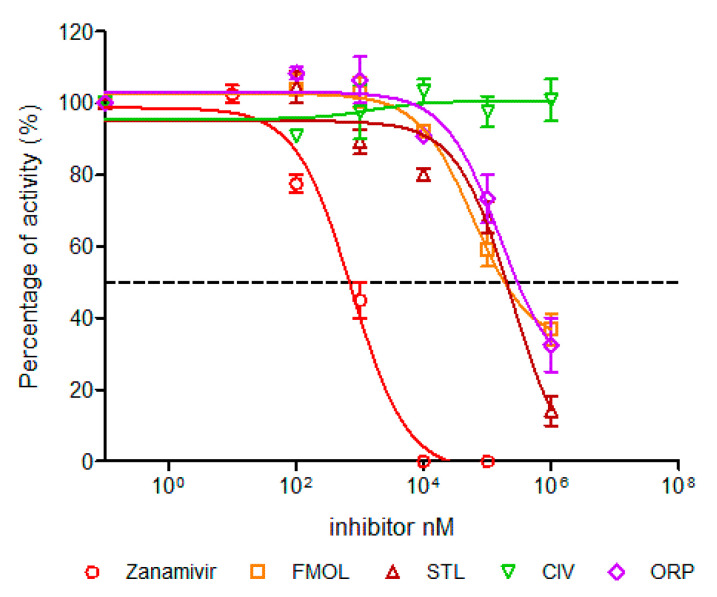
Concentration-activity diagram and corresponding IC_50_ values of viral replication inhibition. IC_50_ was calculated using GraphPad Prism 6.04. Nonlinear regression analysis was performed to obtain trend lines. Data represent the average ± SD of three independent experiments. The experiment with the molecule AAMOL was not carried out. The color code for the diagram lines of NA inhibition is as follows: red corresponds to zanamivir, orange FMOL, brown STL, green CIV and purple ORP.

The inhibition of viral replication was evaluated in MDCK cell cultures by means of lytic plaques for reference zanamivir as well as FMOL, STL, CIV and ORP (Figure 8). To observe their effects under reproducible in vitro test conditions, the amount and size of lytic plaques in presence of the test substances were considered [51,52,53]. Zanamivir inhibits viral replication in concentrations of 1.0 to 10 μM [52,54]. At 10 μM or higher lytic plaque formation was not detected. For the test substances of AAMOL and CIV no inhibition effect was observed at concentrations of 1 μM. For the inhibition control zanamivir a higher concentration was needed to inhibit viral replication in cell culture (μM) than to inhibit the neuraminidase activity (nM). In contrast, FMOL, STL and ORP inhibited viral replication in culture in the millimolar order (cf. IC_50_ in Figure 9).

AAMOL and ORP inhibited enzymatic activity competitively, whereas CIV showed a non-competitive mechanism under millimolar assay conditions. FMOL affected the size and number of plaques at concentrations of 1 mM and 100 μM. STL completely inhibited at concentrations of 1 mM, losing this effect when diluted to 100 μM. ORP only lowered the number of plaques at a concentration of 1 mM, but when the concentration was even more lowered it finally lost its inhibitory drug action (Figure 9, Table 2). The detrimental effects on living cells were also evaluated and the numeric outcome of the standard cytotoxicity test was listed in detail in Supplementary Materials (Table S1). CC_50_ is the drug concentrations necessary to reduce cytotoxicity or cell viability by 50%. The CC_50_ values of ORP were observed in millimolar ranges, which allows us to use higher concentrations without toxic effects, likewise, AAMOL. Its cytotoxicity is lower, since it is metabolized over time as it is a compound based on two amino acids. Both substances constitute optimal candidates to develop new antiviral drugs. The other compounds can be modified to generate products with greater antiviral activities, seeking to reduce their toxic effects at the same time.

AAMOL inhibits NA in millimolar concentrations with a competitive inhibition mechanism. It constitutes a monosubstituted dipeptide, hence its LADMET properties could be expected to be favorable, especially cellular uptake, biotransformation and toxicity. Even larger quantities (to compensate the weak affinity) should not cause toxic side effects. The challenge of derivatizations is twofold: not only potency must be improved but also the compound decay by nonenzymatic hydrolysis must be must be delayed by introducing suitable modifications.

ORP imitates zanamivir’s binding mode through the carboxyl group on the ring. The coinciding binding positions of their carboxyl groups and both six-membered rings allow the introduction of functional groups in ring positions that become equivalent to the functionalities of the side chains of zanamivir. In the same manner, reducing the aliphatic chain length of the sulfonyl group should enhance target affinity. As an asset for ORP counts the observation that its receptor affinity was not affected during docking simulations with mutated neuraminidases with respect to wild type NA. The simulated viral mutant types were studied because they cause known drug resistance to oseltamivir and zanamivir [55,56,57].

CIV is the smallest of all five molecules. It inhibits the activity of neuraminidase in a non-competitive way at millimolar concentrations. Its kinetic behavior can be explained by the formation of stable covalent bonds instead of the reversible noncovalent bonding between the other four scaffolds to the receptor residues. Prior to describing the two possible chemical reactions of covalent bond formation by vitamin C it is paramount to understand the following aspects: (i) Its relatedness to reactivity and stereo configurations of monosaccharides, precisely their acid derivatives l-gulonate or 3-oxo-l-gluonate by a twofold dehydration reaction. (ii) Its chemical bond formalism shows keto-enol tautomerism. Only the enol form—and not the keto form—became detectable in solid phase or solution by crystallography or ^13^C-NMR, respectively. The tautomeric preference is due to the strong stabilizing effects of two intramolecular hydrogen bonds between the oxo group of the lactone (R-O-C(R)=O) and both hydroxyl groups of the endiol form (HO-C(R)=C(R)-OH). (iii) It is a bivalent Bronsted acid and its surprising acidity strength is slightly stronger than acetic acid (pKa = 4.76) for its first proteolytic step with a measured pKa value of 4.17 thanks to its vinylogous carboxylate anion formation directly stemming from its preferred endiol form (O=C(R)-C(OH)=C(R)-O^−^ + H^+^). (iv) In aqueous solution it can react as a CH acidic component (>C-H, >C^−^ + H^+^) in Michael addition reactions with acrolein-like agents (R_2_>C=C(R)-C(R)=O). (v) Finally, its prevailing endiol form strongly reduces substances in aqueous redox reactions. During this reaction ascorbic acid is converted into dehydroascorbic acid which shows its essential role in cell physiology as antioxidant [58].

Based on the aforementioned reactivity, our experimentally determined non-competitive mechanism suggests the covalent bond formation for ascorbic acid in either of two ways: (A) after oxidation and under aerobic conditions a hydrolysis of the endocyclic ester takes place in the first step, this means a decay of the lactone ring by a water moiety as an attacking nucleophilic general base. The direct product is 2,3-diketo-l-gulonic acid which is highly reactive (instable) to form new bonds due to its two vicinal “oxo” oxygen atoms (O=C(R)=C(R)=O). The second way (B) is more likely to occur under anaerobic conditions, but also in situations with a relative absence of molecular oxygen (O_2_). At the initial stage, the enol form of ascorbic acid is converted into its unusual keto form (O=C(R)-C(=O)-C(R)-OH) leading to a multi-step degradation to produce furfural but also other cyclic or ring-opened aldehydes (R-C(H)=O are conceivable. The resulting carbonyl function again is highly reactive to produce more stable adducts under covalent bond formation. Potential counterparts for reaction (A) are the basic amino acids histidine and arginine or the neutral asparagine and glutamine, as well as aromatic tyrosine and tryptophan. On the other side, almost all side chains of the amino acids are amenable to covalent bond formation via (B), except for aliphatic amino acids, proline, methionine or glycine. Of note, traces of heavy metal cations—typically copper or iron ions—catalyze the decay of vitamin C, too. Intriguingly, the latter metal constitutes the central atom at the biocatalytic site of ubiquitarian ascorbic acid oxidase.

Thanks to its obviously irreversible blocking mechanism in addition to its already established biochemical effects as a vitamin, it could be used as a comedication for treatment. Higher dosage would be needed to compensate for its very weak potency. To circumvent its poor target affinity, future research work could explore the chemical space of its favorable derivatives. Upon administration along with neuraminidase inhibitors they could jointly achieve a synergistic effect. Fortunately, CIV offers many possibilities for structural modification to achieve more interaction at the active site. Another promising molecular feature is that thanks to its smaller size and molecular weight (176 Daltons), it effectively possesses multiple binding modes. During our simulations it interacted with different residues in the binding cavity. It lends the opportunity to explore new locations and pockets, too. CIV can generate a larger variety of hydrogen bond networks than all other scaffolds under study here. As a downside, in its present non-substituted form, it is not capable of efficiently occupying the binding cavity. Interestingly, derivatives of ascorbic acid with pentacyclic triterpene extensions have been reported [59,60]. They interact with viral hemagglutinin, inhibiting the entry of the virus into the cell. Considering its noncompetitive inhibition mechanism, this small organic molecule (SOM) can be used with other compounds to work synergistically.

## 3. Discussion

Five non-sialic acid-like compounds are proposed as unsubstituted lead compounds with scaffolds whose likelihood of inherited viral resistance to NA inhibitors is fairly reduced because all five do not constitute analogs to established drugs tightly imitating sialic acid. The novel scaffolds only loosely resemble the natural substrate sialic acid at a pharmacological cost of target affinity and subsequent antiviral potency. All docked solutions of the five molecules were analyzed to establish their binding modes and compare them to the crystal structures of reference drugs. Yet, the proposed structures constitute either undecorated or poorly decorated (“under-decorated”) scaffolds providing the opportunity to follow two opposing research and development strategies: (i) to follow the known substitution of ring positions of zanamivir or oseltamivir and other commercial drugs, which is a straight-forward method used to find stabilizing effects for successful binding by imitating the drugs’ binding modes with the downside of introducing a potentially detrimental risk of suffering inherited drug resistance from virus strains of clinical relevance. (ii) To explore new substitution patterns to improve receptor affinity and at the same time reduce susceptibility to existing influenza A resistance mechanisms against existing commercial drugs thanks to the establishment of hitherto unseen binding patterns.

The present work introduces opportunities to position either new or “borrowed” functional groups on new scaffolds which will help optimize the energetically favorable concert of hydrogen bonding, salt bridges, polar and nonpolar interactions. The five unusual scaffolds will occupy new areas and pockets at the binding site to reach micro or nanomolar drug potency. For instance, in future studies, ORP derivatives could be tested with reduced number of carbon atoms on its aliphatic chains. Hence, steric hindrance will be reduced at the active site. Like CIV, ORP certainly requires the rearrangement of -OH groups to achieve an optimized receptor interaction. In contrast, polar substituents “borrowed” from commercial drugs could be introduced into its basic structure too. In more general terms, a kind of chimeric “recycling” effort could reuse the substituents seen on zanamivir as well as oseltamivir.

## 4. Materials and Methods

The PDB entries were retrieved from the RCSB Protein Data Bank (RCSB PDB) [61]. The complete amino acid sequences of the viral neuraminidase were selected according to Prachanronarong [36] using the Influenza Virus Database (https://www.ncbi.nlm.nih.gov/genomes/FLU) [62]. The primary sequences were aligned through ClustalW, a web-based multiple sequence alignment tool (https://www.ebi.ac.uk/Tools/msa/clustalw2/) [63].

### 4.1. Molecular Docking

Molecular docking is a suitable technique for generating and labeling protein–ligand complexes according to their binding affinities. In this study, molecular docking was performed using the program PrPx-Phyton v0.8 [20,21]. The proposed non-sialic acid like structures, zanamivir and sialic acid were docked to the catalytic site of two NA subtypes in a search area of 70 Å × 60 Å × 60 Å with an exhaustivity of 1000. Only the computed complexes with the best binding energies were taken to generate 2D diagrams by means of the LigPlot v.1.4.5 [24]. The surface representation of the protein with the bound ligand was carried out under UCSF Chimera 1.11.2 [64] using its Coulombic Surface Coloring tool. For molecular docking, two NA structures with PBD codes 3B7E [22] and 4GZW [23] were used. The literature attests that both possess a high percentage of identification to the consensus sequences of neuraminidase subtypes N1 and N2, respectively [24]. Neuraminidase of A/Tanzania/205/2010 H3N2 virus presented 94% identity and 96% similarity to the consensus sequence which had been determined in MSA studies of 8745 complete sequences. In contrast, neuraminidase of A/Brevig Mission/1/1918 H1N1 virus showed 92% identity along with a 96% similarity to a consensus sequence which was determined from 7370 aligned sequences. As a most valuable asset, the studied crystal structures of subtype N1 have a fairly high crystallographic resolution (≤2.0 Å). Moreover, at the active site, the aforementioned Loop 150 is resolved in those N1 crystal structures [24].

Prior to docking, the two missing viral neuraminidase structures of NA Puerto Rico H1N1 (A/PR/8/34) and NA Chicken México H5N2 (A/Chicken/Mexico/31381-7/1994) were generated from their sequences of amino acids in the I-TASSER platform [30,31]. Precisely, I-TASSER applies a local meta-threading approach (LOMETS [34]) to conduct the initial sequence alignments. This step is followed by a search for 3D templates. To generate the target structure models I-TASSER uses the TM-align structural alignment program to match the first 3D model to all other template structures that it can find among the PDB entries. For generating the 3D homology models of both missing target structures, the following PDB codes were used: 4QN3, 2AEP, 3TIA, 6N4D and 6BR5 for NA Puerto Rico H1N1, as well as 4QN3, 3NSS, 5HUN, 2HT5, 5NWE and 2HTV for NA Chicken México H5N2, respectively. The model validation at the same website gave us no hints about bad or unusual geometrical features.

In the next step, both neuraminidase models were prepared for docking under UCSF Chimera 1.11.2 and Vega ZZ 3.1.1.42 [65,66]. To this end, one monomer was selected and hydrogen atoms were added at physiological pH (7.4). Crystal water and other moieties that remained from the stage of crystallogenesis were detected and eliminated.

The five proposed SOMs, sialic acid and reference drug zanamivir were prepared in the program Avogadro 1.2.0 [67]. For these seven structures the protonation–dissociation states were determined at physiological pH. Their geometries were optimized using the MMFF94 force field in this program [68].

Finally, the 3D target models were inspected for completeness and the monomeric target structures were used for blind docking of all five SOMs. Sialic acid and zanamivir were included as references for re-docking studies and to compare the docked poses and computed data with their crystallographically known binding modes and measured activities [25]. Docking was performed in a grid box of 70 Å × 60 Å × 60 Å dimensions, i.e., a space which completely covers the active site of the NA.

### 4.2. Cells and Viral Strains

Influenza A/Puerto Rico/8/1934 (H1N1) (A/PR/8/34) virus was obtained from the American Type Culture Collection (ATCC). Influenza A/Chicken/México/31381-7/1994 (H5N2) was donated by A. Aguilar Setien. Madin-Darby canine kidney (MDCK) cells (ATCC, Manassas, VA, UAS) were maintained in Dubelco Eagle’s Minimum Essential Medium supplemented with 5% fetal bovine serum and a mixture of antibiotics and antimycotics (100 U of penicillin, 0.1 mg of streptomycin, and 0.25 μg of amphotericin B/mL). The supernatants of the infected cultures were obtained after 96 h post-infection, clarified by centrifugation at low speed and stored at −70 °C for further use. Viral inoculum was titrated through the formation of lytic plaques in the same cell line using the conventional method in medium solidified with 0.5% agarose and stained with violet crystal [69]. Titers were reported in plaque forming units per milliliter (PFU/mL).

### 4.3. Small Organic Molecules

The five proposed SOMs are structurally unrelated to each other (Figure 1). They were tested for inhibition of the viral neuraminidase activity by in vitro bioassays as well as cell culture experiments. The rational chemical denominations and internally used acronyms (nick names) of the molecules are as follows: propanoic acid 3-[(2,5-dimethylphenyl) carbamoyl]-2-(piperazin-1-yl) (FMOL) and *N*-acetylphenylalanylmethionine (AAMOL) and 3-(propyl aminosulfonyl)-4-chlorobenzoic acid (STL, “TL” alludes to its donator) were all donated by Prof. Dr. Thierry Langer, University Vienna. Ascorbic acid (CIV) and 4-(dipropylsulfamoyl) benzoic acid (ORP) were donated by Dr. Stefan Kahlert, Institute of Anatomy, Otto von Guericke University Magdeburg, Magdeburg, Germany. The compounds were dissolved in distilled water and aliquots were stored at −20 °C for later usage.

### 4.4. Measurement of NA Activities

The NA activities of both influenza A viruses (H1N1 and H5N2) were measured by a fluorescence-based assay using the fluorogenic substrate MUNANA (Sigma-Aldrich, St Louis, MO, USA), based on the method of Potier et al. 1979 [70,71,72]. Substrate cleavage by the enzyme NA releases the fluorescent product 4-methylumbelliferone (4-MU) (Sigma-Aldrich). Fluorescence was measured every 60 s for 60 min at 37 °C in a Synergy 2 multimode microplate reader (BioTek Instruments, Winooski, VT, USA), using excitation and emission wave lengths of 360 nm and 460 nm. Two-fold virus dilutions were prepared in enzyme buffer (32.5 mM of 2-(*N*-morpholino) ethanesulfonic acid, AKA MES, 4 mM of calcium chloride, pH 6.5) and added (100 mL/well) in duplicate to a flat-bottom 96-well opaque black plate (Corning, Tewksbury, MA, USA). The MUNANA substrate was added to all wells (50 mL/well) to achieve a final concentration of 100 µM. Immediately after adding the MUNANA substrate, the plate was transferred to a prewarmed Synergy 2 multimode microplate reader at 37 °C. The fluorescence signal from the enzyme buffer and MUNANA substrate alone in the absence of enzyme was treated as background noise and as a direct consequence, was subtracted from the signals obtained in the other wells. A standard curve was generated for each experiment using 4-MU diluted in enzyme buffer at final concentrations of 0.05 to 200 µM [73].

### 4.5. NA Enzyme Kinetics Assay

NA enzymatic parameters were determined in reactions in flat-bottom 96-well opaque black plates (Corning, Tewksbury, MA, USA). The necessary influenza virus preparations were described above, and various MUNANA substrate concentrations were also prepared. All assay components were pre-warmed for 20–30 min at 37 °C. The reaction was conducted at 37 °C in a total volume of 150 µL. In the next step, the fluorescence of the released 4-methylumbelliferone was measured during 20 min incubation. The reaction finished by the addition of the stop solution (0.1 M glycine in 25% ethanol, pH 10.7). Fluorometric determinations were quantified with a Synergy2 multi-mode microplate reader (BioTek Instruments, Winooski, VT, USA) using excitation and emission wave lengths of 360 and 460 nm, respectively [73]. The kinetic data were fitted to the Michaelis-Menten equation by using nonlinear regression (GraphPad Prism 6.04; GraphPad, San Diego, CA, USA) to determine the Michaelis constant (Km) and maximum velocity (Vmax) of substrate conversion.

IC_50_ values from NA were determined in reactions performed as described above. The five molecules AAMOL, FMOL, STL, CIV and ORP were used in different concentrations. The reference drug zanamivir was used as inhibition control. Prior to testing, all assay components were warmed up at 37 °C for 20 min. The test reaction was conducted at 37 °C in a total volume of 150 µL, and the fluorescence of the released 4-methylumbelliferone was measured after 20 min incubation time. The reaction was stopped upon addition of 0.1 M glycine in 25% ethanol at pH 10.7. Fluorometric determinations were quantified with a Synergy 2 multi-mode microplate reader (BioTek Instruments, Winooski, VT, USA) using excitation and emission wavelengths of 360 and 460 nm, respectively. The data were fitted using nonlinear regression (GraphPad Prism 6.04) to determine the IC_50_ values for the molecules [48,74,75,76].

### 4.6. Toxicity Assays

The substances of the five proposed scaffolds were tested on MDCK cells cultured in 96-well plates following the MTT colorimetric method. Briefly, 20,000 cells per well were incubated with several concentrations (1 nM–75mM) for 24 and 48 h. After that, the medium was removed, cells were washed twice with PBS, 100 μL of 0.5 mg/mL MTT was added into each well and incubated for 3 h at 37 °C. After that, 100 μL of 0.1% DMSO was added and the optical density was determined at 570 nm. A standard curve was included with known amounts of untreated cells. The percentage of cytotoxicity was calculated by comparing treated versus untreated cells, the 50% cytotoxic concentration (CC_50_) was calculated using GraphPad Prism 6.04 [77].

### 4.7. Statistical Analysis

Data analyses were performed and plotted in the GraphPad Prism software version 6.0 (GraphPad Software, La Jolla, CA, USA). Statistical analysis was performed using one-way ANOVA and Bonferroni test. *p*-values under the threshold of *p* < 0.001 were considered statistically significant.

## 5. Conclusions

Five small organic compounds which do not resemble either each other or any of the hitherto known commercial drugs—but show promising results of in silico docking simulation results—were tested for their in vitro inhibitory potency against viral neuraminidases of human and avian influenza A viruses (H1N2 and H5N2). All five scaffolds demonstrated weak inhibition activity. Ligand docking at the known binding sites of wild type and mutant type NAs lent insight into potential binding patterns at an atomic scale. The computed binding modes for all five ligands could be brought on an equal footing with the measured activities as well as the competitive or non-competitive inhibition mode. The advantages and disadvantages of binding of each scaffold was discussed in details. The detected disadvantages lend opportunities for improvements. Hence all five are proposed here as lead compounds for future drug development cycles to strengthen target selectivity and specificity. Precisely, the missing functional groups of zanamivir and oseltamivir offer opportunities to not only follow the example of their substitution patterns, but also attach new functional groups to contribute to the hitherto unseen binding modes to circumvent existing drug resistance at the binding site. All told, we present five unusual scaffolds with experimentally determined activity to inhibit neuraminidases in the millimolar range which can be used to develop new oral drugs against influenza A.

## Figures and Tables

**Table 1:** Inhibition constant (K_i_) and half maximal inhibitory concentration (IC_50_) of molecules against influenza A virus strain. For the FMOL and STL molecules, only the IC_50_ with the avian subtype H5N2 was determined.

Molecule	Influenza A Virus Strain			
	A/PR/18/34 H1N1		A/CHK/MX/1994 H5N2	
	Ki (mM)	IC50 (mM)	Ki (mM)	IC50 (mM)
AAMOL	15.3	57 ± 1.1 *	24.37	73 ± 1.18 *
CIV	26.43	32 ± 1.5 *	17.03	17.5 ± 1.2 *
ORP	10.5	23 ± 1.2 *	3.4	15.3 ± 1.13 *
FMOL	ND ^1^	ND ^1^	ND ^1^	6.4 ± 1.1 *
STL	ND ^1^	ND ^1^	ND ^1^	24.8 ± 1.3 *
Zanamivir	2.58 × 10 ^−6^	1.19 × 10^−6^ ± 0.043	1.59 × 10^−5^	2.84 × 10^−6^ ± 0.047

^1^ ND: not determined. * *p* < 0.001 compared with the respective controls.

**Table 2:** IC_50_ of molecules against influenza A virus strain A/CHK/MX/1994 H5N2.

Molecule	Influenza A Virus Strain A/CHK/MX/1994 H5N2
	IC50 (mM)
AAMOL	ND
FMOL	0.059 ± 1.28 *
STL	0.243 ± 1.59 *
CIV	-
ORP	0.157 ± 1.50 *
Zanamivir	7.41 × 10^−4^ ± 1.24

ND: not determined. * *p* < 0.001 compared with the respective controls.

## Supplementary Materials

The following are available online. Table S1: Cytotoxicity of molecules on MDCK cells for 24 and 48 h following the MTT colorimetric method.

## References

[1] Dawood F.S., Iuliano A.D., Reed C., Meltzer M.I., Shay D.K., Cheng P.Y., Bandaranayake D., Breiman R.F., Brooks W.A., Buchy P. (2012). Estimated global mortality associated with the first 12 months of 2009 pandemic influenza A H1N1 virus circulation: A modelling study. Lancet Infect. Dis..

[2] Iuliano A.D., Roguski K.M., Chang H.H., Muscatello D.J., Palekar R., Tempia S., Cohen C., Gran J.M., Schanzer D., Cowling B.J. (2018). Estimates of global seasonal influenza-associated respiratory mortality: A modelling study. Lancet.

[3] Bouvier N.M., Palese P. (2008). The biology of influenza viruses. Vaccine.

[4] Hay A.J., Gregory V., Douglas A.R., Lin Y.P. (2001). The evolution of human influenza viruses. Philos. Trans. R. Soc. Lond. B Biol. Sci..

[5] Carter J.B., Saunders V.A. (2007). Virology: Principles and Applications.

[6] Palese P S.M. (2007). Orthomyxoviridae: The viruses and their replication. Fields Virol..

[7] von Itzstein M., Wu W.Y., Kok G.B., Pegg M.S., Dyason J.C., Jin B., Van Phan T., Smythe M.L., White H.F., Oliver S.W. (1993). Rational design of potent sialidase-based inhibitors of influenza virus replication. Nature.

[8] Li W., Escarpe P.A., Eisenberg E.J., Cundy K.C., Sweet C., Jakeman K.J., Merson J., Lew W., Williams M., Zhang L. (1998). Identification of GS 4104 as an orally bioavailable prodrug of the influenza virus neuraminidase inhibitor GS 4071. Antimicrob. Agents Chemother..

[9] Colman P.M. (1994). Influenza virus neuraminidase: Structure, antibodies, and inhibitors. Protein Sci..

[10] Taylor N.R., von Itzstein M. (1994). Molecular modeling studies on ligand binding to sialidase from influenza virus and the mechanism of catalysis. J. Med. Chem..

[11] Gamblin S.J., Skehel J.J. (2010). Influenza hemagglutinin and neuraminidase membrane glycoproteins. J. Biol. Chem..

[12] Russell R.J., Haire L.F., Stevens D.J., Collins P.J., Lin Y.P., Blackburn G.M., Hay A.J., Gamblin S.J., Skehel J.J. (2006). The structure of H5N1 avian influenza neuraminidase suggests new opportunities for drug design. Nature.

[13] Wang P., Zhang J.Z. (2010). Selective binding of antiinfluenza drugs and their analogues to ‘open’ and ‘closed’ conformations of H5N1 neuraminidase. J. Phys. Chem. B.

[14] Lackenby A., Besselaar T.G., Daniels R.S., Fry A., Gregory V., Gubareva L.V., Huang W., Hurt A.C., Leang S.K., Lee R.T.C. (2018). Global update on the susceptibility of human influenza viruses to neuraminidase inhibitors and status of novel antivirals, 2016–2017. Antivir. Res..

[15] Toledo-Rueda W., Rosas-Murrieta N.H., Munoz-Medina J.E., Gonzalez-Bonilla C.R., Reyes-Leyva J., Santos-Lopez G. (2018). Antiviral resistance markers in influenza virus sequences in Mexico, 2000–2017. Infect. Drug Resist..

[16] Scior T., Bender A., Tresadern G., Medina-Franco J.L., Martinez-Mayorga K., Langer T., Cuanalo-Contreras K., Agrafiotis D.K. (2012). Recognizing pitfalls in virtual screening: A critical review. J. Chem. Inf. Model..

[17] Colman P.M., Hoyne P.A., Lawrence M.C. (1993). Sequence and structure alignment of paramyxovirus hemagglutinin-neuraminidase with influenza virus neuraminidase. J. Virol..

[18] Wang M., Qi J., Liu Y., Vavricka C.J., Wu Y., Li Q., Gao G.F. (2011). Influenza A virus N5 neuraminidase has an extended 150-cavity. J. Virol..

[19] Gubareva L.V., Robinson M.J., Bethell R.C., Webster R.G. (1997). Catalytic and framework mutations in the neuraminidase active site of influenza viruses that are resistant to 4-guanidino-Neu5Ac2en. J. Virol..

[20] Dallakyan S., Olson A.J. (2015). Small-molecule library screening by docking with PyRx. Methods Mol. Biol..

[21] Trott O., Olson A.J. (2010). AutoDock Vina: Improving the speed and accuracy of docking with a new scoring function, efficient optimization, and multithreading. J. Comput. Chem..

[22] Xu X., Zhu X., Dwek R.A., Stevens J., Wilson I.A. (2008). Structural characterization of the 1918 influenza virus H1N1 neuraminidase. J. Virol..

[23] Zhu X., McBride R., Nycholat C.M., Yu W., Paulson J.C., Wilson I.A. (2012). Influenza virus neuraminidases with reduced enzymatic activity that avidly bind sialic Acid receptors. J. Virol..

[24] Wallace A.C., Laskowski R.A., Thornton J.M. (1995). LIGPLOT: A program to generate schematic diagrams of protein-ligand interactions. Protein Eng..

[25] Malbari K.D., Chintakrindi A.S., Ganji L.R., Gohil D.J., Kothari S.T., Joshi M.V., Kanyalkar M.A. (2019). Structure-aided drug development of potential neuraminidase inhibitors against pandemic H1N1 exploring alternate binding mechanism. Mol. Divers..

[26] Finley J.B., Atigadda V.R., Duarte F., Zhao J.J., Brouillette W.J., Air G.M., Luo M. (1999). Novel aromatic inhibitors of influenza virus neuraminidase make selective interactions with conserved residues and water molecules in the active site. J. Mol. Biol..

[27] Hsu K.C., Hung H.C., Horng J.T., Fang M.Y., Chang C.Y., Li L.T., Chen I.J., Chen Y.C., Chou D.L., Chang C.W. (2013). Parallel screening of wild-type and drug-resistant targets for anti-resistance neuraminidase inhibitors. PLoS ONE.

[28] Anuwongcharoen N., Shoombuatong W., Tantimongcolwat T., Prachayasittikul V., Nantasenamat C. (2016). Exploring the chemical space of influenza neuraminidase inhibitors. PeerJ.

[29] Collins P.J., Haire L.F., Lin Y.P., Liu J., Russell R.J., Walker P.A., Skehel J.J., Martin S.R., Hay A.J., Gamblin S.J. (2008). Crystal structures of oseltamivir-resistant influenza virus neuraminidase mutants. Nature.

[30] Roy A., Kucukural A., Zhang Y. (2010). I-TASSER: A unified platform for automated protein structure and function prediction. Nat. Protoc..

[31] Yang J., Yan R., Roy A., Xu D., Poisson J., Zhang Y. (2015). The I-TASSER suite: Protein structure and function prediction. Nat. Methods.

[32] Maring C.J., Stoll V.S., Zhao C., Sun M., Krueger A.C., Stewart K.D., Madigan D.L., Kati W.M., Xu Y., Carrick R.J. (2005). Structure-based characterization and optimization of novel hydrophobic binding interactions in a series of pyrrolidine influenza neuraminidase inhibitors. J. Med. Chem..

[33] Stoll V., Stewart K.D., Maring C.J., Muchmore S., Giranda V., Gu Y.G., Wang G., Chen Y., Sun M., Zhao C. (2003). Influenza neuraminidase inhibitors: Structure-based design of a novel inhibitor series. Biochemistry.

[34] Luo M. (2006). Structural biology: Antiviral drugs fit for a purpose. Nature.

[35] Varghese J.N., Colman P.M., van Donkelaar A., Blick T.J., Sahasrabudhe A., McKimm-Breschkin J.L. (1997). Structural evidence for a second sialic acid binding site in avian influenza virus neuraminidases. Proc. Natl. Acad. Sci. USA.

[36] Prachanronarong K.L., Özen A., Thayer K.M., Yilmaz L.S., Zeldovich K.B., Bolon D.N., Kowalik T.F., Jensen J.D., Finberg R.W., Wang J.P. (2016). Molecular basis for differential patterns of drug resistance in influenza N1 and N2 neuraminidase. J. Chem. Theory Comput..

[37] Liu Z., Zhao J., Li W., Wang X., Xu J., Xie J., Tao K., Shen L., Zhang R. (2015). Molecular docking of potential inhibitors for influenza H7N9. Comput. Math. Methods Med..

[38] Starkey I.D., Mahmoudian M., Noble D., Smith P.W., Cherry P.C., Howes P.D., Sollis S.L. (1995). Synthesis and influenza virus sialidase inhibitory activity of the 5-desacetamido analogue of 2,3-didehydro-2,4-dideoxy-4-guanidinyl-N-acetylneuraminic acid (GG167). Tetrahedron Lett..

[39] Smith P.W., Starkey I.D., Howes P.D., Sollis S.L., Keeling S.P., Cherry P.C., von Itzstein M., Wu W.Y., Jin B. (1996). Synthesis and influenza virus sialidase inhibitory activity of analogues of 4-guanidino-Neu5Ac2en (GG167) with modified 5-substituents. Eur. J. Med. Chem..

[40] Shie J.J., Fang J.M., Lai P.T., Wen W.H., Wang S.Y., Cheng Y.S., Tsai K.C., Yang A.S., Wong C.H. (2011). A practical synthesis of zanamivir phosphonate congeners with potent anti-influenza activity. J. Am. Chem. Soc..

[41] Feng E., Shin W.-J., Zhu X., Li J., Ye D., Wang J., Zheng M., Zuo J.-P., No K.T., Liu X. (2013). Structure-based design and synthesis of C-1- and C-4-modified analogs of zanamivir as neuraminidase inhibitors. J. Med. Chem..

[42] Kongkamnerd J., Milani A., Cattoli G., Terregino C., Capua I., Beneduce L., Gallotta A., Pengo P., Fassina G., Miertus S. (2012). A screening assay for neuraminidase inhibitors using neuraminidases N1 and N3 from a baculovirus expression system. J. Enzym. Inhib. Med. Chem..

[43] Smith P.W., Sollis S.L., Howes P.D., Cherry P.C., Starkey I.D., Cobley K.N., Weston H., Scicinski J., Merritt A., Whittington A. (1998). Dihydropyrancarboxamides related to zanamivir: A new series of inhibitors of influenza virus sialidases. 1. Discovery, synthesis, biological activity, and structure-activity relationships of 4-guanidino- and 4-amino-4H-pyran-6-carboxamides. J. Med. Chem..

[44] Das A., Adak A.K., Ponnapalli K., Lin C.H., Hsu K.C., Yang J.M., Hsu T.A., Lin C.C. (2016). Design and synthesis of 1,2,3-triazole-containing N-acyl zanamivir analogs as potent neuraminidase inhibitors. Eur. J. Med. Chem..

[45] Wyatt P.G., Coomber B.A., Evans D.N., Jack T.I., Fulton H.E., Wonacott A.J., Colman P., Varghese J. (2001). Sialidase inhibitors related to zanamivir. Further SAR studies of 4-amino-4H-pyran-2-carboxylic acid-6-propylamides. Bioorg. Med. Chem. Lett..

[46] Lin C.-H., Chang T.-C., Das A., Fang M.-Y., Hung H.-C., Hsu K.-C., Yang J.-M., von Itzstein M., Mong K.K.T., Hsu T.-A. (2013). Synthesis of acylguanidine zanamivir derivatives as neuraminidase inhibitors and the evaluation of their bio-activities. Org. Biomol. Chem..

[47] Barnett J.M., Cadman A., Gor D., Dempsey M., Walters M., Candlin A., Tisdale M., Morley P.J., Owens I.J., Fenton R.J. (2000). Zanamivir susceptibility monitoring and characterization of influenza virus clinical isolates obtained during phase II clinical efficacy studies. Antimicrob. Agents Chemother..

[48] Wetherall N.T., Trivedi T., Zeller J., Hodges-Savola C., McKimm-Breschkin J.L., Zambon M., Hayden F.G. (2003). Evaluation of neuraminidase enzyme assays using different substrates to measure susceptibility of influenza virus clinical isolates to neuraminidase inhibitors: Report of the neuraminidase inhibitor susceptibility network. J. Clin. Microbiol..

[49] Marathe B.M., Leveque V., Klumpp K., Webster R.G., Govorkova E.A. (2013). Determination of neuraminidase kinetic constants using whole influenza virus preparations and correction for spectroscopic interference by a fluorogenic substrate. PLoS ONE.

[50] Govorkova E.A., Leneva I.A., Goloubeva O.G., Bush K., Webster R.G. (2001). Comparison of efficacies of RWJ-270201, zanamivir, and oseltamivir against H5N1, H9N2, and other avian influenza viruses. Antimicrob. Agents Chemother..

[51] Weight A.K., Haldar J., Alvarez de Cienfuegos L., Gubareva L.V., Tumpey T.M., Chen J., Klibanov A.M. (2011). Attaching zanamivir to a polymer markedly enhances its activity against drug-resistant strains of influenza a virus. J. Pharm. Sci..

[52] Baer A., Kehn-Hall K. (2014). Viral concentration determination through plaque assays: Using traditional and novel overlay systems. J. Vis. Exp..

[53] Hatakeyama S., Sakai-Tagawa Y., Kiso M., Goto H., Kawakami C., Mitamura K., Sugaya N., Suzuki Y., Kawaoka Y. (2005). Enhanced expression of an alpha2,6-linked sialic acid on MDCK cells improves isolation of human influenza viruses and evaluation of their sensitivity to a neuraminidase inhibitor. J. Clin. Microbiol..

[54] Matrosovich M., Matrosovich T., Garten W., Klenk H.D. (2006). New low-viscosity overlay medium for viral plaque assays. Virol. J..

[55] Samson M., Pizzorno A., Abed Y., Boivin G. (2013). Influenza virus resistance to neuraminidase inhibitors. Antivir. Res..

[56] Kode S.S., Pawar S.D., Tare D.S., Keng S.S., Hurt A.C., Mullick J. (2019). A novel I117T substitution in neuraminidase of highly pathogenic avian influenza H5N1 virus conferring reduced susceptibility to oseltamivir and zanamivir. Vet. Microbiol..

[57] Choi W.S., Jeong J.H., Kwon J.J., Ahn S.J., Lloren K.K.S., Kwon H.I., Chae H.B., Hwang J., Kim M.H., Kim C.J. (2018). Screening for neuraminidase inhibitor resistance markers among avian influenza viruses of the N4, N5, N6, and N8 neuraminidase subtypes. J. Virol..

[58] Little K., Leang S.K., Butler J., Baas C., Harrower B., Mosse J., Barr I.G., Hurt A.C. (2015). Zanamivir-resistant influenza viruses with Q136K or Q136R neuraminidase residue mutations can arise during MDCK cell culture creating challenges for antiviral susceptibility monitoring. Eurosurveillance.

[59] Roth H.J., Eger K., Troschütz R. (1981). Pharmazeutische Chemie II: Arzneistoffanalyse-Reaktivität, Stabilität, Analytik.

[60] Brinkevich S.D., Boreko E.I., Savinova O.V., Pavlova N.I., Shadyro O.I. (2012). Radical-regulating and antiviral properties of ascorbic acid and its derivatives. Bioorg. Med. Chem. Lett..

[61] Wang H., Xu R., Shi Y., Si L., Jiao P., Fan Z., Han X., Wu X., Zhou X., Yu F. (2016). Design, synthesis and biological evaluation of novel L-ascorbic acid-conjugated pentacyclic triterpene derivatives as potential influenza virus entry inhibitors. Eur. J. Med. Chem..

[62] Berman H.M., Westbrook J., Feng Z., Gilliland G., Bhat T.N., Weissig H., Shindyalov I.N., Bourne P.E. (2000). The protein data bank. Nucleic Acids Res..

[63] Brister J.R., Ako-Adjei D., Bao Y., Blinkova O. (2015). NCBI viral genomes resource. Nucleic Acids Res..

[64] Hall T.A. (1999). Bioedit: A user-friendly biological sequence alignment editor and analysis program for windows 95/98/NT. Nucleic Acids Symp. Ser..

[65] Pettersen E.F., Goddard T.D., Huang C.C., Couch G.S., Greenblatt D.M., Meng E.C., Ferrin T.E. (2004). UCSF Chimera—A visualization system for exploratory research and analysis. J. Comput. Chem..

[66] Batool S., Mushtaq G., Kamal W., Kamal M.A. (2016). Pharmacophore-based virtual screening for identification of novel neuraminidase inhibitors and verification of inhibitory activity by molecular docking. Med. Chem..

[67] Neri-Bazan R.M., Garcia-Machorro J., Mendez-Luna D., Tolentino-Lopez L.E., Martinez-Ramos F., Padilla M., Aguilar-Faisal L., Soriano-Ursua M.A., Trujillo-Ferrara J.G., Fragoso-Vazquez M.J. (2017). Design, in silico studies, synthesis and in vitro evaluation of oseltamivir derivatives as inhibitors of neuraminidase from influenza A virus H1N1. Eur. J. Med. Chem..

[68] Hanwell M.D., Curtis D.E., Lonie D.C., Vandermeersch T., Zurek E., Hutchison G.R. (2012). Avogadro: An advanced semantic chemical editor, visualization, and analysis platform. J. Cheminform..

[69] Halgren T.A. (1996). Merck molecular force field. I. Basis, form, scope, parameterization, and performance of MMFF94. J. Comput. Chem..

[70] Racaniello V.R., Palese P. (1979). Isolation of influenza C virus recombinants. J. Virol..

[71] Potier M., Mameli L., Belisle M., Dallaire L., Melancon S.B. (1979). Fluorometric assay of neuraminidase with a sodium (4-methylumbelliferyl-alpha-D-N-acetylneuraminate) substrate. Anal. Biochem..

[72] Leang S.K., Hurt A.C. (2017). Fluorescence-based Neuraminidase Inhibition Assay to Assess the Susceptibility of Influenza Viruses to The Neuraminidase Inhibitor Class of Antivirals. J. Vis. Exp..

[73] Mungall B.A., Xu X., Klimov A. (2003). Assaying susceptibility of avian and other influenza A viruses to zanamivir: Comparison of fluorescent and chemiluminescent neuraminidase assays. Avian Dis..

[74] Tu V., Abed Y., Barbeau X., Carbonneau J., Fage C., Lague P., Boivin G. (2017). The I427T neuraminidase (NA) substitution, located outside the NA active site of an influenza A(H1N1)pdm09 variant with reduced susceptibility to NA inhibitors, alters NA properties and impairs viral fitness. Antivir. Res..

[75] Govorkova E.A., Ilyushina N.A., McClaren J.L., Naipospos T.S., Douangngeun B., Webster R.G. (2009). Susceptibility of highly pathogenic H5N1 influenza viruses to the neuraminidase inhibitor oseltamivir differs in vitro and in a mouse model. Antimicrob. Agents Chemother..

[76] Yen H.L., Ilyushina N.A., Salomon R., Hoffmann E., Webster R.G., Govorkova E.A. (2007). Neuraminidase inhibitor-resistant recombinant A/Vietnam/1203/04 (H5N1) influenza viruses retain their replication efficiency and pathogenicity in vitro and in vivo. J. Virol..

[77] Eisenbrand G., Pool-Zobel B., Baker V., Balls M., Blaauboer B.J., Boobis A., Carere A., Kevekordes S., Lhuguenot J.C., Pieters R. (2002). Methods of in vitro toxicology. Food Chem. Toxicol.

