# Supplementary material for: Five Novel Non-Sialic Acid-Like Scaffolds Inhibit In Vitro H1N1 and H5N2 Neuraminidase Activity of Influenza a Virus
Source: Molecules. 2020 Sep 16;25(18):4248. doi: 10.3390/molecules25184248 (PMC7571124; doi:10.3390/molecules25184248)
Supplement: Supplementary file 1 [file molecules-25-04248-s001.pdf]

Supplementary Materials for

Five Novel Non-Sialic Acid-Like Scaffolds Inhibit In Vitro H1N1 and H5N2 Neuraminidase Activity of Influenza A Virus

Table S1. Cytotoxicity of molecules on MDCK cells for 24 and 48 hours following the MTT colorimetric method.

**Molecule**	**24 h**	**48 h**
	CC_{50} (μM)	
AAMOL	16.0 ± 1.5	ND
FMOL	0.11 ± 1.3	10.2 ± 1.4
STL	1.41 ± 1.3	1.38 ± 1.3
CIV	0.3 ± 1.2	0.55 ± 1.1
ORP	6110 ± 1.1	1189 ± 1.3

ND: not determined.
